# Buffalo Medical and Surgical Association

**Published:** 1891-11

**Authors:** W. S. Renner

**Affiliations:** Secretary


### BUFFALO MEDICAL AND SURGICAL ASSOCIATION.

Reported by W. S. RENNER, M. D., Secretary.

*Stated Meeting, September 1st.*

THE PRESIDENT being absent, Dr. BARTLETT occupied the chair.

Dr. A. E. PERSONS read a paper entitled, Disorders of Nutrition (continued).

DISCUSSION.

Dr. VAN PEYMA thought that most professional men were inclined to be lazy. We require more exercise to keep our digestive organs in repair.

Dr. A. L. BENEDICT does not consider washing out the stomach a curative agent; it is like washing the external surface of the body; it cleanses the stomach, the same as a bath washes the skin.

Dr. COAKLEY was glad to hear the reader emphasize "diet" in the treatment of stomach troubles, both in regard to its quality and quantity; much can often be attained by restricting the diet of a patient; many cases present peculiarities, some patients are better on an animal diet, some do best on a vegetable diet, some can take only fluids, others only solids. He thinks exercise is very important in these cases; it stimulates normal tissue changes; it should be taken in the open air; horseback riding is the best kind of exercise: it is exhilarating and the organs are well shaken up and are thereby made to secrete better. He agreed with Dr. Benedict in regard to the benefit arising from washing out the stomach.

Dr. Pryor thinks that lavage in the treatment of the stomach is at present too much of a fad. He believes that *lavage* is useful in chronic catarrh where there is a large amount of mucus, which is preventing the food from coming in contact with the stomach walls and mixing with the gastric juice. He doubts the utility of electricity; it may give more motion to the muscular walls, but cannot reduce the size of a dilated stomach. He thought that Dr. Persons had confused lithemia with neurasthenia, and said that there was a great difference between these two conditions.

Dr. Dunham: Physical exercise, out-door work, and change of occupation often help these cases. He had had under his care a case of general tuberculosis with dilatation of the stomach, in which the condition of the stomach greatly improved within two weeks on five drops of tincture of the perchloride of iron with ten drops of the fluid extract of ergot, three times daily.

Dr. Hayd: Most of the disorders of nutrition are due to imperfect oxidation, thus filling the blood with excrementitious matter; consequently the method of overcoming these disorders is by taking out-door exercise. It naturally follows that we should be very careful what medicines we prescribe, for these conditions are usually found in people of sedentary habits, who become languid early in the day; these people smoke in the house: thus they paralyze the pneumogastric, and produce an atonic condition of the stomach. When people say that lavage simply washes the stomach, they do not appreciate its therapeutic value; it tones up the muscular coat and increases the amount of hydrochloric acid, a fact which has been conclusively proven. It would be as foolish to apply medicines to the unwashed skin as it would be to give medicines in such cases without first washing out the stomach. The cases of dyspepsia which we meet every day do not usually require lavage, but exercise and care in taking food. He said that lithemia differed from neurasthenia, in that the patient with the first condition is extremely irritable.

Dr. Grant said that reflexes often produce disturbances of the stomach. He related a case in which the patient had been troubled with hardness of hearing and cough; this patient vomited every morning. He removed a glass diamond from the child's ear, took it off cough-mixtures, etc. The patient recovered at once from all the symptoms. The symptoms in this case were due to reflex through the small branch of the pneumogastric, which goes to the ear.

Dr. PERSONS said that Dr. Pryor had misunderstood him in regard to lithemia and neurasthenia.

